# Targeting transcription factors for therapeutic benefit in rheumatoid arthritis

**DOI:** 10.3389/fimmu.2023.1196931

**Published:** 2023-06-29

**Authors:** Thivya Balendran, Keith Lim, John A. Hamilton, Adrian A. Achuthan

**Affiliations:** ^1^ Department of Medicine, Royal Melbourne Hospital, The University of Melbourne, Parkville, VIC, Australia; ^2^ Department of Medicine, Western Health, The University of Melbourne, St Albans, VIC, Australia

**Keywords:** transcription factors, rheumatoid arthritis, cytokines, NF-κB, AP-1, STAT and IRF

## Abstract

Rheumatoid arthritis (RA) is a destructive inflammatory autoimmune disease that causes pain and disability. Many of the currently available drugs for treating RA patients are aimed at halting the progression of the disease and alleviating inflammation. Further, some of these treatment options have drawbacks, including disease recurrence and adverse effects due to long-term use. These inefficiencies have created a need for a different approach to treating RA. Recently, the focus has shifted to direct targeting of transcription factors (TFs), as they play a vital role in the pathogenesis of RA, activating key cytokines, chemokines, adhesion molecules, and enzymes. In light of this, synthetic drugs and natural compounds are being explored to target key TFs or their signaling pathways in RA. This review discusses the role of four key TFs in inflammation, namely NF-κB, STATs, AP-1 and IRFs, and their potential for being targeted to treat RA.

## 1 Introduction

Rheumatoid arthritis (RA) is a chronic inflammatory autoimmune disease that mostly affects joints. Joint inflammation is initiated and maintained by a complex interaction between many cells, including T cells, dendritic cells, B cells, macrophages, neutrophils, osteoclasts, and fibroblast-like synoviocytes (FLS) (1). These cells can release pro-inflammatory cytokines, chemokines, reactive oxidative species, matrix metalloproteinases (MMPs) and autoantibodies into synovial joints and thus contribute inflammation, cartilage damage, osteoclast activation, and bone destruction (2–4).

Many pro-inflammatory mediators have been implicated in the pathogenesis of RA (5). For example, tumor necrosis factor (TNF), interferons (IFNs), interleukin (IL) -1β, IL-2, IL-4, IL-6, IL-8, IL-17, IL-18, IL-21, IL-22, IL-23 and granulocyte macrophage-colony stimulating factor (GM-CSF) have been suggested to play a central role in RA pathogenesis (6–8). These cytokines activate key transcription factors (TFs), such as nuclear factor-κB (NF-κB), activator protein-1 (AP-1), interferon regulatory factors (IRFs), and signal transducer and activator of transcription (STAT) proteins, which can further promote the production of pro-inflammatory mediators (9). Therefore, targeting these key TFs or the signaling pathways associated with these TFs is a feasible strategy for treating RA. While several synthetic drugs are currently being trialed aimed at targeting key TFs in RA, several natural compounds have also been explored as potential alternative treatment options with a focus on targeting TFs. In this review, we summarize the role of four families of TFs, namely NF-κB, STATs, AP-1 and IRFs, in the pathogenesis of RA, and provide an update on the latest preclinical and clinical trials targeting them.

## 2 NF-κB

### 2.1 NF-κB signaling pathway

The NF-κB signaling pathway controls many biological processes, but its dysregulation is often associated with inflammation, for example, that associated with RA. Activated NF-κB is observed in RA synovium in early and late stages of joint inflammation and initiation of inflammation is triggered by NF-κB activation in both T cells and antigen presenting cells (10). Different extracellular and/or intracellular stimuli (e.g., TNF, IL-1β, IL-6, MMPs and RANKL) can activate the NF-κB signaling pathway, either directly or indirectly (11). The NF-κB family is composed of five structurally related members that include NF-κB1/p50 (precursor p105), NF-κB2/p52 (precursor p100), RelA/p65, RelB, and c-Rel which bind to specific DNA and κB enhancer elements that mediate the transcription of target genes (12, 13). NF-κB is activated via two different pathways. Direct activation includes canonical and non-canonical pathways, mediated by inhibitor of kappa B (IκB) kinase (IKK) and NF-κB-inducing kinase (NIK), respectively. The indirect activation of NF-κB is interconnected with other cellular pathways, including mitogen-activated protein kinase (MAPK), Rho, and phosphoinositide 3-kinase (PI3-K) (11).

#### 2.1.1 Canonical pathway

**Figure 1:**
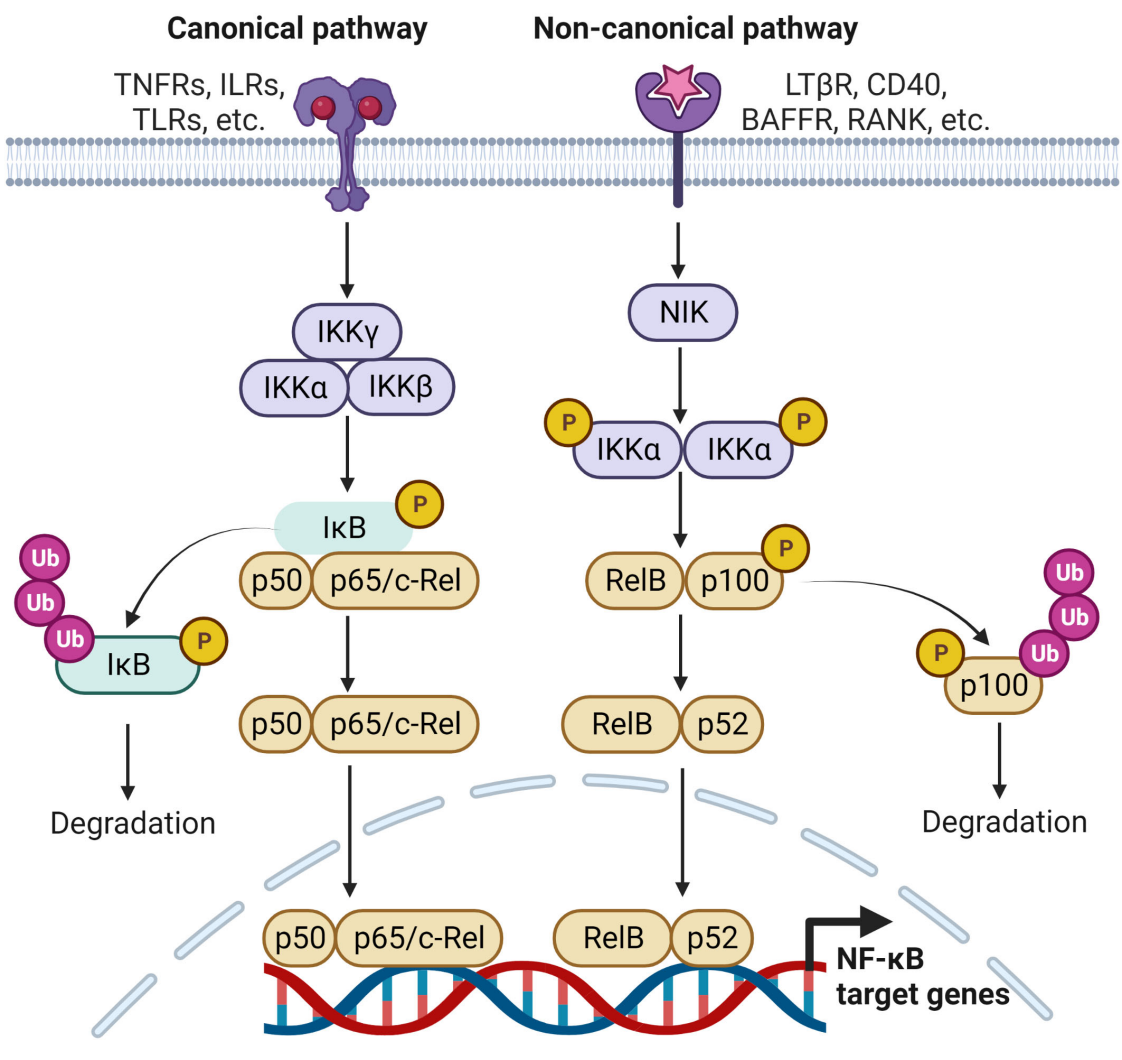
Signaling pathways leading to the regulation of NF-κB target genes. The activation of NF-κB involves two signaling pathways, the canonical and non-canonical pathways. Both are activated through engagement with distinct receptors, leading to transcriptional regulation several NF-κB target genes that are responsible for immune and inflammatory responses.

In inflammatory conditions, such as in RA, cytokines, chemokines and free radicals provide signals that lead to degradation of IκB protein resulting in the disassociation of NF-κB (12, 13) (**Figure 1**). Activation of the canonical pathway occurs through stimulation of the TNF receptors, Toll-like receptors (TLRs), interleukin receptors, pattern recognition receptors (PRRs), T cell receptors (TCRs) and B cell receptors (BCRs) (14, 15). The canonical pathway has an IKK complex, comprising IKKα and IKKβ, the homologous catalytic subunits, and IKKγ, a regulatory subunit of the complex that activates IKKβ (14). Receptor activation stimulates numerous kinases, such as transforming growth factor-β (TGF-β)-activated kinase 1 (TAK1), receptor-interacting protein kinase 1 (RIP1), MAPK kinase ERK1 (MEKK1) and TANK-binding kinase (TBK1), which phosphorylate IKKβ and activate the IKK complex (16). Activated IKKβ then phosphorylates IκBα and activates its downstream TFs, RelA/p50 and p50/c-Rel (13). The liberated RelA/p50 and p50/c-Rel translocate to the nucleus and activate the transcription of NF-κB-dependent inflammatory genes (14, 17).

#### 2.1.2 Non-canonical pathway

Noncanonical NF-κB pathway respond to a certain type of stimulus, such as the lymphotoxin β receptor (LTβR), CD40, the B-cell activating factor receptor (BAFFR) and receptor activator of NF-κB (RANK) (18) (**Figure 1**). NIK is essential for the activation of this pathway and is central to the signaling that activates IKKα and forms a functional cooperation with IKKα to phosphorylate p100. Phosphorylation of p100 stimulates the partial proteasomal processing of p52 (11). This generates NF-κB2/p52 through the degradation of the p100 C-terminal IκB-like structure, and leads to the nuclear translocation of p52/RelB occurs (19).

### 2.2 NF-κB-regulated genes

NF-κB regulates more than 150 genes involved in anti-apoptosis, cell proliferation, immunity, and inflammation. It plays a key role in regulating the activation, survival, and differentiation of innate and adaptive immune cells (20). In RA, a deregulated NF-κB signaling pathway contributes to the pathogenic process and activates both immune and non-immune cells (e.g., FLS) through transcriptional regulation of inflammatory mediators, including TNF, IL-1, IL-2, IL-6, IL-8, IL-9, IL-12, IL-18, IL-23, GM-CSF, VEGF, RANKL, MCP-1, MIP-2, CXCL1, CXCL10, RANTES, ICAM-1, VCAM-1, MMPs, and COX-2. (10, 13, 20, 21). These NF-κB-regulated inflammatory mediators have been reported to play a crucial role in the pathogenesis of RA by activating both immune and non-immune cells.

T cells and macrophages are key responders to the NF-κB signaling pathway. Deregulated NF-κB signaling causes aberrant activation of T cells and each member of the NF-κB family is responsible in activating different types of T cells in RA. RelA and c-Rel activate naïve T cells by inducing TCR activation. c-Rel promotes the transcription of Foxp3, a key regulator of Tregs (22). NF-κB differentiates Th1 and Th17 cells by inducing IL-12 production and promotes IL-17 synthesis in Th17 cells, and thereby recruiting neutrophils and monocytes to sites of inflammation. Th17 cells contribute to inflammation by regulating expression of TNF, IL-1β, IL17, IL-21, and IL-22 (23). Noncanonical NF-κB regulates Th17 to induce GM-CSF. On the other hand, Th2 responses are regulated by NF-κB1/p50. In macrophages, NF-κB induces a range of inflammatory mediators, including TNF, IL-1β, IL-6, IL-12, and COX-2. Activated c-Rel is essential for IL-12B expression and also for NF-κB-ATF3-CEBPδ transcriptional circuit, which enables macrophages to analyze the responses received from persistent and transient TLR4 stimulation (24). In FLS, NF-κB p50/p52 and NFATc1 respond to RANKL and exhibit an inflammatory response along with osteoclast activation and osteoclast genesis (18). NFATC1 is a major TF that regulates osteoclast differentiation (25). Together with NFATC1, RelB regulates osteoclast formation (26). Given the broad range of inflammatory roles of NF-B, its targeting might be beneficial for treating RA.

### 2.3 Current treatments targeting NF-κB

#### 2.3.1 Synthetic drugs

Conventional disease-modifying antirheumatic drugs (cDMARDs) and biological DMARDs (bDMARDs) have been used to treat RA for many decades. Methotrexate (MTX) is a first-line drug widely used to treat RA, while bDMARDs, such as TNF inhibitors, have been used since 1980. Currently, five main classes of TNF-inhibiting bDMARDs are available: etanercept, adalimumab, certolizumab pegol, golimumab, and infliximab. A recent clinical trial suggests switching from TNF inhibitors to tacrolimus (TAC) after acquiring low disease activity. TAC is an immunosuppressant that can block the calcineurin pathway in T cells by inhibiting cytokine production and T cell proliferation (27). Artemisinin-type compounds inhibit several receptor-coupled signaling pathways that include IL-1, TNF, RANKL, growth factor receptors, and TLRs (4). Terfenadine and Fexofenadine have recently been identified as more cost-effective and safer TNF inhibitors (28). Regulation of RANKL levels is maintained by bDMARDs (e.g., Denosumab) (29). All the above-mentioned drugs target cytokines that can activate NF-κB, thereby indirectly suppressing its activity. The long-term use of these drugs and the need to increase the dosage for an effective result can lead to adverse effects, such as osteoporosis, hyperlipidemia, hepatitis, tuberculosis, malignancies, and adrenal insufficiency (30). Furthermore, there is an increase in resistance to these drug in 30% of cases of RA (31).

Since there is a need for a different approach to reduce side effects, recent studies focus directly on targeting NF-κB, thus potentially achieving more precision in treating RA (**Table 1**). Tetrandrine, a bisbenzylisoquinoline, blocks NF-κB/RelA (32). Iguratimod is a new synthetic targeted DMARD (stDMARD) that inhibits the translocation of NF-κB to the nucleus and is approved only in China and Japan for RA treatment (33). Small-interfering RNA (siRNA) targeting NF-κB, delivered in combination with MTX inside a liposome capsule, prevents its release in the circulation, avoiding possible adverse effects of MTX (17). Chen et al., have demonstrated that low molecular weight polyethyleneimine cholesterol polyethylene glycol encapsulates siRNA as an efficient way to silence NF-κB/p65 to restore an anti-inflammatory microenvironment in RA (34). Drug delivery via nanocarriers is now being explored to deliver controlled doses of drug of interest to promote cell/tissue specific treatment, thus minimizing the potential side effects (43).

**Table 1:** Synthetic drugs and natural compounds targeting NF-κB either directly or indirectly.

Target	Drugs	Effects on NF-κB-regulated inflammatory factors	Study type	Reference(s)
Synthetic drugs				
NF-κB	Tetrandrine	Inhibits IL-1β, TNF and IL-6	Clinical trial NCT05245448	(32)
NF-κB	Iguratimod	Inhibits prostaglandin E2, bradykinin, IL-1β, IL-6, IL-8, GM-CSF, TNF and COX-2	Clinical trial NCT03855007	(33)
NF-κB	siRNA	Inhibits IL-1, TNF, IFNγ and IL-6 production	In vitro	(17, 34)
Natural compounds				
NF-κB	Vitamin D	Inhibits RANK, CXCL10, and IL-17a.	Clinical trial NCT04344405	(18)
NF-κB	Celastrol	Inhibits IL-1β, TNF, substance P, β-endorphin, MMP9, COX-2, c-Myc, TGF-β, c-JUN, JAK1, JAK3, IKKβ, SYK, MMP3 and MEK1.	In vitro	(31, 35–37)
NF-κB	Curcumin	Inhibits IL-1, TNF, and IL-6. Increases IL-10	In vitro	(38, 39)
NF-κB	Resveratrol	Inhibits COX-2, iNOS, TNF, MMP3, MMP13	In vitro	(40, 41)
NF-κB	Quercetin	Inhibits IL-1β, IL-6, IL-8, IL-13, TNF and IL-17	In vitro	(42)

#### 2.3.2 Natural compounds

To minimize side effects caused by synthetic drugs, many studies are now focusing on natural compounds that can alleviate RA disease (**Table 1**). Celastrol, triptolide, resveratrol, curcumin, myricetin, fisetin and quercetin have been identified to hopefully reduce RA severity by targeting numerous cytokines, signaling pathways and proteases (41, 44). Numerous studies have shown the effect of celastrol on actively improving RA severity through suppression of the following: ROS-NF-κB-NLRP3 signaling (37), HIF expression and ROS release (36), the PI3-K/AKT/mTOR axis (45, 46) and NF-κB by degrading IκB (44, 47). Resveratrol, a polyphenol, activates sirt1, which suppresses the transcriptional activity of NF-κB/p65 by deacetylation and inhibits the COX/MMP pathway and the production of IL-1β, IL-6, and TNF (48). Curcumin suppresses the expression of NF-κB by upregulating that of miR-124 (39). Emerging findings suggest that treating RA patients with vitamin D supplementation can lower RANKL and CXCL10 levels, and suppress activation of NF-κB (18). Glucosamine prevents the demethylation of particular CpG sites in the promotor region of IL-1β, thereby preventing NF-κB from binding to the promotor region and suppressing the expression of IL-1β (49). These studies indicate the potential of natural compounds to not only target NF-κB, but also to suppress inflammation in RA.

## 3 JAK/STAT

### 3.1 JAK/STAT signaling pathway

**Figure 2:**
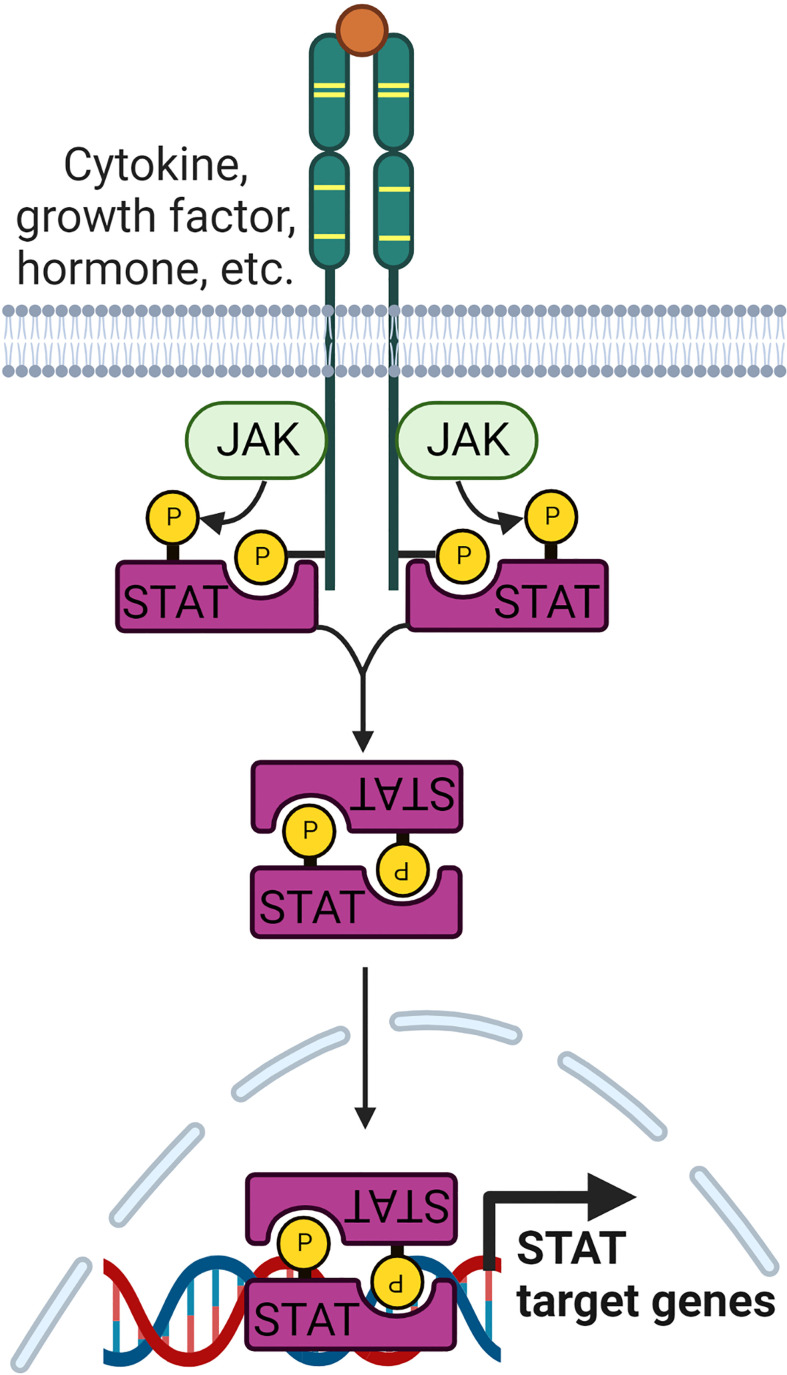
Signaling pathway leading to the regulation of JAK/STAT target genes. The family of JAK kinases are activated upon receptor ligation. Subsequently, they recruit STAT family proteins and phosphorylate them. Phosphorylated STATs form homo or heterodimers before being translocated to the nucleus to regulate transcription of STAT target genes.

The Janus kinase/signal transducer and activator of transcription (JAK/STAT) pathway is a key signaling pathway important in governing many biological processes, including cell differentiation, proliferation, and immune functions. Several studies have identified that the JAK/STAT signaling pathway is deregulated in RA (50, 51). Many of the proinflammatory cytokines, including TNF, IL-β, IL-6, IL-7, IL-8, IL-12, IL-15, IL-17, IL-23, IL-32, IFN and GM-CSF, that are highly expressed in RA are known to be regulated by JAK/STAT signaling pathway (50, 52). The JAK family has four members, JAK1, JAK2, JAK3 and tyrosine kinase 2 (TYK2), while the STAT family of TFs consists of seven members, namely STAT1, STAT2, STAT3, STAT4, STAT5a, STAT5b, and STAT6. Upon receptor ligation, JAKs are autophosphorylated, and recruit and phosphorylate members of the STAT family (**Figure 2**). Phosphorylated STATs dissociate from the receptor and form homo or heterodimers before translocating to the nucleus to activate the transcription of STAT-regulated genes (53). STATs bound to gene promoters can be dephosphorylated by nuclear protein tyrosine phosphatases (N-PTPs) and subsequently exit the nucleus to the cytoplasm for further activation cycles (54). Negative regulators of the JAK/STAT pathway, such as PTPs, protein inhibitors of activated STAT (PIAS), and suppressors of cytokine signaling proteins (SOCS), play crucial roles in controlling STAT-regulated gene expression (55–57).

JAKs and STATs are activated by stimulation with various cytokines (51, 58). JAK1 is phosphorylated by four types of cytokine receptor families: (i) cytokine receptor with γ_c_ (IL-2, IL-4, IL-7, IL-9, IL-15, and IL-21 receptors); (ii) receptors with gp130 subunits (IL-6, IL-11, IL-27, oncostatin M (OSM), cardiotrophin-1 (CT-1), leukemia inhibitory factor (LIF), cardiotrophin-like cytokine (CLC), and ciliary neurotrophic factor (CNTF) receptors; (59) (iii) class 2 cytokine receptors (IL-10 family, type 1 and 2 IFN receptors); and (iv) IL-3, IL-5, and GM-CSF receptors. As for JAK1, JAK2 is activated by (i) the gp130 receptor family, (ii) the IL-3R family (IL-3, IL-5R and GM-CSF receptors), (iii) the class 2 cytokine receptor family, and (iv) single chain receptors, such as growth hormone, thrombopoietin, prolactin and erythropoietin receptors. JAK3 is activated by IL-2, IL-4, IL-7, IL-9, IL-15 and IL-21 receptors and TYK2 is activated by IFN type 1, IL-6 family, IL-10 family, IL-12, IL-13, and IL-23 receptors (60).

STAT1 is activated by IFNγ, IL-2, IL-6, IL-7, IL-21, epidermal growth factor (EGF), platelet-derived growth factor (PDGF), TNF, hepatocyte growth factor (HGF) and angiotensin 2. It has been found that IFNα/β are the only cytokines that can activate STAT2. STAT3 is activated by the IL-6 cytokine family (IL-6, IL-11, IL-27, IL-31, CNTF, OSM, and LIF), the IL-10 cytokine family (IL-10, IL-19, IL-20, IL-22, IL-24, IL-26, IL-28A, IL-28B and IL-29), GM-CSF, IL-2, IL-7, IL-21, IFNα/β, and leptin. STAT4 is activated by IL-12, IL-23 and IFNα/β whereas STAT5 is activated by the IL-3, the IL-2 cytokine family (IL-2, IL-4, IL-7, IL-9, IL-15 and IL-21), prolactin, EGF, GM-CSF, PDGF and GH. STAT6 is activated only by IL-4 and IL-13 (58, 61, 62).

### 3.2 STAT-regulated genes

STATs bind directly to DNA regulatory elements and regulate gene transcription. STAT1, STAT3, STAT4, STAT5, and STAT6 have been shown to be highly expressed in RA (63). STATs often interact with other TFs, which assemble in the promotor or enhancer regions of target genes. Examples of such interactions are STAT1-STAT2 with IRF9, STAT1 with NF-κB, STAT3 with Jun, STAT3 with IRF4, RORγt, and BAFT in T cells, and STAT1 with IRF1, IRF8 and PU.1 in macrophages. These complex transcription networks highlight the fact that multiple TFs can be involved in regulating gene expression in various cell types.

#### 3.2.1 STAT1

Various STAT proteins play a different role in different cell types that lead to RA pathogenesis. In synovial macrophages, STAT1 activates CXCL9 and CXCL10, which recruit T cells, induce Th1 differentiation, and upregulate IFNγ production (64, 65). STAT1 activation is essential for activated IRF1 and TLR3 in macrophages (66). STAT1 can induce iNOS expression and produce NO, which can reduce cell migration, while suppressing STAT3 activity (67). Most importantly, STAT1 regulates MMP3 and MMP13, thereby inducing cartilage degradation in the knee joint (68). A study by Kuuliala et al. has suggested that the activation of STAT1 and STAT6 in circulating leukocytes helps predict the response to treatment in RA (69).

#### 3.2.2 STAT3

STAT3 is another major TF involved in inflammation. STAT3 induces angiogenesis, transcription of B cell lymphoma protein 2 (BCL-2), several MMPs, including MMP1, 3, and 13, and cyclins (70, 71). STAT3 activates RORγt, which induces IL-6 production and leads to Th17 polarization and stabilization (72). It can inhibit fibroblast apoptosis (73), the function of STAT1, and the expression of IFNα (74).

#### 3.2.3 STAT4, STAT5, and STAT6

The production of the Th1-driven cytokine, IL-12, is mediated through STAT4. STAT5 promotes the production of CD4^+^ T cells (75). A few studies have reported the role of STAT5 in GM-CSF-induced CCL17 production (76, 77), a chemokine found to be important in inflammatory arthritis (78). IL-4 transduces signal through STAT6, which regulates the Treg cell response (55, 69).

### 3.3 Current treatments targeting JAK/STAT

#### 3.3.1 Synthetic drugs

JAK/STATs are key regulators of cytokines produced in RA pathogenesis and therefore, are considered as feasible drug targets (60). Currently, JAK inhibitors (JAKi) are used as third-line therapy for RA patients with disease recurrence after using MTX and bDMARDs. JAKi are tsDMARDs, and they competitively inhibit by binding to the ATP binding site of the kinase domain present in JAKs, thereby inhibiting the JAK phosphorylation and preventing STAT activation (60). Among the currently available JAKi, baricitinib and tofacitinib are pan JAKi (**Table 2**). Baricitinib, tofacitinib and upadacitinib are approved by the FDA for the treatment of RA (87), while filgotinib and peficitinib are being evaluated (82). Tofacitinib is highly selective for JAK1 and JAK3, with less selectivity for JAK2 and TYK2 (64). Baricitinib inhibits JAK1 and JAK2, while moderately inhibits TYK2 and JAK3 (88). One study showed a similar safety profile for baricitinib and tofacitinib, but a better clinical outcome with baricitinib (89). The introduction of a nanostructure-based drug delivery system enables site-specific delivery of tofacitinib and the JNK inhibitor SP600125 (90). Upadacitinib and filgotinib selectively inhibit JAK1 and have been proven to be efficient in phase 2 and 3 studies (91). Another study demonstrated that baricitinib combined with MTX and upadacitinib with MTX can effectively inhibit the JAK/STAT signaling pathway (82).

**Table 2:** Synthetic drugs and natural compounds targeting JAK/STAT either directly or indirectly.

Target	Drugs	Effects on JAK/STAT-regulated inflammatory factors	Study type	Reference(s)
Synthetic drugs				
JAK1/3	Tofacitinib	Inhibits STAT1, STAT3, STAT5, CXCL9, and CXCL10	FDA-approved	(64, 79)
JAK1/2	Baricitinib	Inhibits IL-6, IL-12, IL-23, IFNγ, CXCL9 and CXCL10	FDA-approved	(73)
JAK1	Upadacitinib	–	FDA-approved	(80)
JAK1	Fligotinib	Inhibits STAT1 and STAT5	FDA-approved	(81)
JAK3	Peficitinib	–	FDA-approved	(82)
Natural compounds				
JAK2/3	Notopterol	Inhibits STAT5	In vitro	(83)
JAK2	Genkwanin	Inhibits STAT3	In vitro	(84)
JAK1/2	Kaempferol	Inhibits STAT1 and STAT3	In vitro	(85)
STAT1/3	EGCG	Inhibits iNOS and ICAM-1	In vitro	(86)

#### 3.3.2 Natural compounds

Although JAKi function effectively in RA patients, they are expensive for broader application and demonstrate adverse effects, including hepatotoxicity, gastrointestinal perforations, thromboembolism, herpes zoster, and tuberculosis; therefore, some studies are focusing on exploring natural compounds that can inhibit JAK/STAT signaling (87) (**Table 2**). Notopterol is a natural compound that effectively inhibits JAK2/JAK3 and suppresses the production of CXCL2, CXCL9, CXCL10, CXCL12, CCL5, IL-1β, IL-6, and TNF levels in bone marrow-derived macrophages. Genkwanin, a flavone, inhibits the JAK/STAT pathway by binding to JAK2 and NF-κB, reducing TNF, NO and IL-6 levels, while increasing IL-10 production (84, 92). Quercetin, epigallocatechin-3-gallate (EGCG), resveratrol, curcumin, genistein, chlorogenic acid, swertiamarin, cyanidin, ferulic acid, baicalein, falcarindiol, cinnamaldehyde and cryptotanshinone have been found to be effective in inhibiting JAK/STATs.

## 4 AP-1

### 4.1 Activation of AP-1

**Figure 3:**
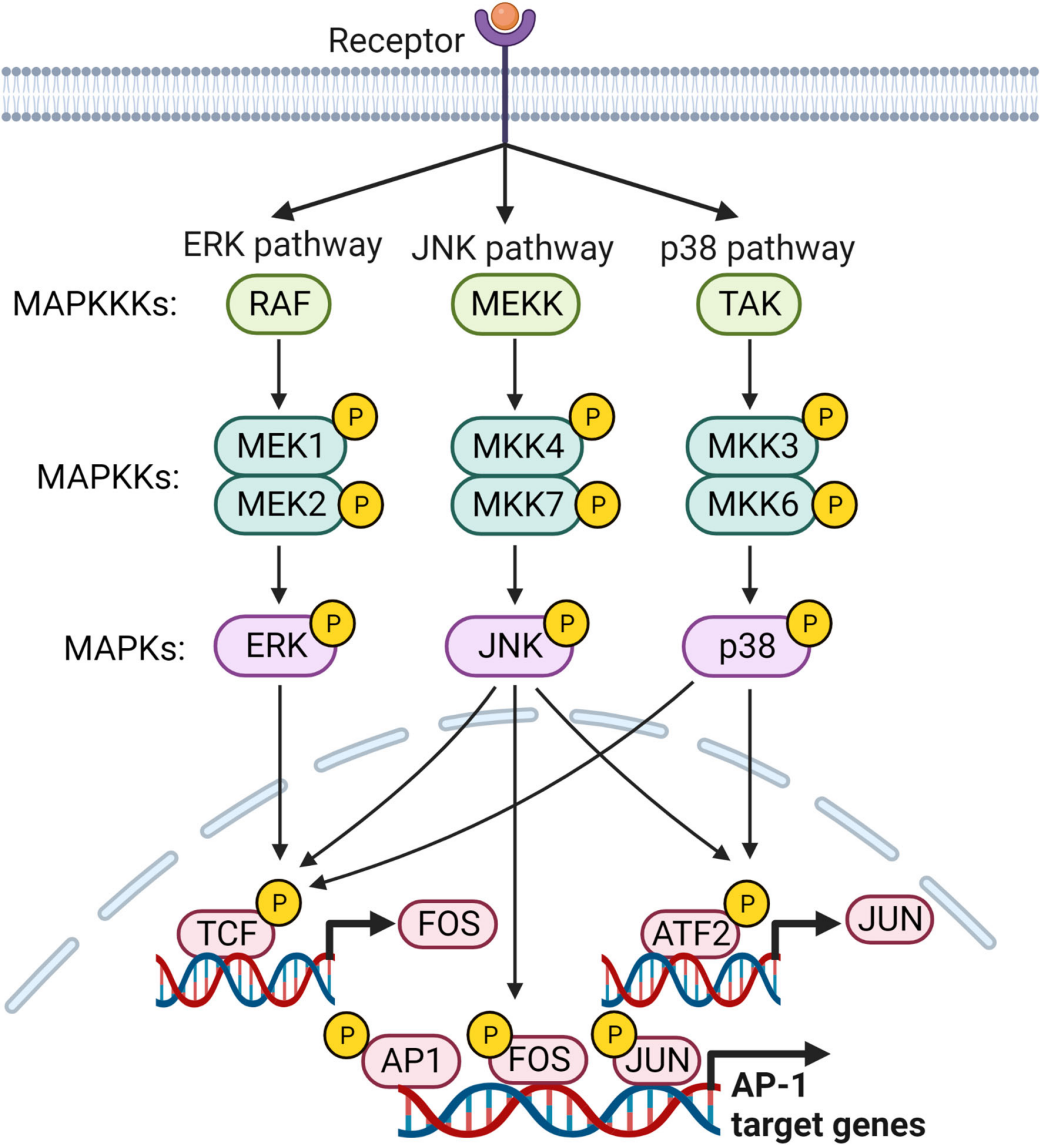
MAP kinase signaling pathways leading to the regulation of AP-1 target genes. Members of MAP kinases, ERK, JNK and p38, are responsible for activating transcription factors that regulate transcription of FOS and JUN genes and their subsequent activation. Subsequently, activated AP-1 binds to the promoter regions of its target genes and regulates their expression.

The activator protein-1 (AP-1) is proposed to play an important role in inflammation and pathogenesis of RA (93). Increased levels of c-Fos and c-Jun in RA synovium are correlated with disease severity (94). In the initial phase of RA, ROS activated AP-1, but in the late phase, proinflammatory cytokines can upregulate AP-1 (95). It is a leucine zipper TF composed of Fos, Jun, and ATF families of proteins (96). Fos proteins (FosB, Fra-1, Fra-2, c-Fos) heterodimerize with members of the Jun family, whereas Jun proteins (c-Jun, JunB and JunD) can heterodimerize and/or homodimerize with members of the Fos family to form transcriptionally functional complexes that bind to the promotor region of AP-1 sites (97) (**Figure 3**). The dimer composition of AP-1 and the active state of the Jun and Fos components determine the target of AP-1 (98). Jun : Jun and Fos : Jun dimers selectively bind to AP-1 motifs, known as the 12-O-tetra-decanoylphorbol-13-acetate (TPA) responsive element (TRE) and the cAMP-responsive element (CRE) (99).

AP-1 is activated primarily by MAPK signaling. The three main subfamilies in the MAPK signaling pathway, extracellular signal-regulated kinases (ERKs), p38, and c-Jun N-terminal kinase (JNK), are essential for activation of AP-1 (96). MAPKs are activated by a cascade of phosphorylation events, wherein activated mitogen-activated protein kinase kinase kinase (MAPKKK) phosphorylates mitogen-activated protein kinase kinase (MAPKK), which finally phosphorylates MAPK (100). IL-1β, IL-6, TNF, TGF-β, and TPA up-regulates AP-1 through the MAPK pathway (101, 102). ERK1/2 is activated through a signaling cascade via phosphorylation of Ras, Raf, and MEK 1/2 (98). Stimulation of TLR4, IL-1R, and TNFR activates MyD88 and TAK1, which activates MKK4/7 or MKK3/6, thereby activating c-Fos/c-Jun of AP-1 by JNK and p38, respectively (103, 104). CXCL1 induces c-Jun phosphorylation in RA synovial fibroblasts (RASFs), and increased activation of AP-1 is observed in CXCL1 treated cells (93). AP-1 is activated by enhanced PI3K/AKT activation through stimulation of TNF- and thrombin-induced EGFR transactivation in chondrocytes (105, 106). Another study in RASFs showed that myostatin-induced TNF expression through the PIK3-AKT-AP-1 signaling pathway by activating the c-Jun binding site found in the TNF gene promoter region (107). Activating transcription factor 2 (ATF2), a member of the AP-1 TF family, is highly expressed in RA FLS activated via ERK and MAPK. Sprouty2 can inhibit ATF2 overexpression by inhibiting the phosphorylation of ERK and MAPK (108).

### 4.2 AP-1-regulated genes

#### 4.2.1 Fos

AP-1 selectively regulates a range of cytokines, chemokines, proteinases, and TFs. Each member of the AP-1 family differentially regulates genes. c-Fos/AP-1 induce the expression of MMPs (e.g., MMP1, 2, 3, 8, 9, and 13) and cytokines (e.g., IL-23) (109, 110). MMPs are mainly regulated by IL-1β-induced c-Fos/AP-1; most genes in the MMP family have an AP-1 binding site in the promoter regions near the TATA box and a mutation in the AP-1 binding site completely suppresses MMP expression (111). MMPs are essential for cartilage joint matrix breakdown and MMP13 predominantly degrades cartilage by cleaving type 2 collagen (111). IL-1β can induce osteoclast genesis directly and/or indirectly through RANKL signaling. Integration of RANKL and M-CSF signaling requires Fos/AP-1 (112).

#### 4.2.2 Jun

c-Jun differentially regulates cyclooxygenase-2 (COX-2) and arginase-1 (ARG-1) and promotes macrophage activation, thus contributing to arthritis progression (113). JunB can control Th17 differentiation by inducing the expression of RORγt and RORα, while suppressing the expression of Foxp3 (72). JunB synergizes with c-MAF and GATA3 and induces activation of IL-4, which induces Th2 cell differentiation (114). cJun and JunB together activate AKT1 by binding directly to its promoter region (115).

#### 4.2.3 NFAT

The nuclear factor of activated T cells (NFAT) is suggested to play a role in the pathogenesis of RA (116). AP-1 interacts with NFAT and cooperatively forms an AP-1/NFAT complex, which enhances transcriptional activity compared to Fos-Jun or NFAT binding and regulates most cytokines. It regulates IL-2, which is required for Treg proliferation (114). As AP-1 regulates some important inflammatory mediators that promote RA, it serves as a treatment target to alleviate RA. Synthetic drugs and natural compounds targeting AP-1 are being studied at present.

### 4.3 Current treatments targeting AP-1

#### 4.3.1 Synthetic drugs

Given the role of AP-1 in the regulation of key inflammatory mediators known to promote RA, targeting it is a potential treatment solution; however, there are no FDA-approved AP-1 inhibitors available in the clinic. Many *in vitro* and *in vivo* studies are currently focusing on drugs that can inhibit AP-1 (**Table 3**). CKD-506 is an orally administered hydroxamate that blocks the activation of AP-1 and NF-κB transcription in peripheral blood mononuclear cells isolated from RA patients (117). T-5224, a molecular inhibitor of c-Fos/AP-1, inhibits the DNA binding of c-Fos/c-Jun, thus inhibiting IL-1β, IL-6, TNF, MMP1, 3, and 13. N-(3-acetamidophenyl)-2-[5-(1H-benzimidazol-2-yl) pyridin2-yl] sulfanylacetamide can disrupt the interaction between AP-1 and NFAT and blocks the transcription of IL-2 and some cyclosporin A-sensitive cytokines (126). A cyclin-dependent kinase 4/6 (CDK) inhibitor (CDKi) blocks AP-1 transcription by decreasing Jun stability, thus blocking the production of MMP3 and attenuating cartilage destruction in the collagen-induced arthritis model (119). A novel JNK inhibitor, 11H-indeno[1,2-b] quinoxaline-11-one oxime, has been shown to not only inhibit JNK phosphorylation but also block the transcriptional activity of AP-1 and NF-κB (120). Roflumilast, a selective phosphodiester-4 inhibitor, inhibits the production of IL-1β, IL-6, TNF, CCL5, CXCL9, CXCL10, MMP3, and MMP13 by blocking the transcriptional activity of AP-1 and NF-κB (118). Many of these synthetic drugs targeting AP-1 show promise in preclinical studies, but further research and clinical trials are needed before obtaining FDA approval.

**Table 3:** Synthetic drugs and natural compounds targeting AP-1 either directly or indirectly.

Target	Drugs	Effects on AP-1-regulated inflammatory factors	Study type	Reference(s)
Synthetic drugs				
**AP-1**	CKD-506	Inhibits TNF, IL-6, IL-8, MMP1, and MMP3	Clinical trial NCT04204603	(117)
**AP-1**	Roflumilast	Inhibits CCL5, CXCL9, CXCL10, MMP3 and MMP13	*In vivo*	(118)
**c-Fos**	T-5224	Inhibits MMP1, 3, 13, TNF, IL-6, and IL-1β	*In vivo*	(111)
**Jun**	CDKI	Inhibits MMP1 and MMP3 production via AP-1 signaling pathway	*In vitro*	(119)
**JNK**	11H-indeno[1,2-b] quinoxalin-11-one oxime	Inhibits IL-6 production by inhibiting AP-1 and NF-κB pathway	*In vitro*	(120)
Natural compounds				
**AP-1**	Thymoquinone	Inhibits TNF and IL-6	*In vivo*	(121)
**AP-1**	Actin K	Inhibits VCAM-1	*In vitro*	(122)
**AP-1**	Apigenin-4^´^-O-α-L-rhamnoside	Inhibits MMP1, MMP3, RANKL and TNF	*In vitro*	(102)
**AP-1**	Thymoquinone	Inhibits ICAM-1, VCAM-1, MAPK, MMP3, MMP13, and COX-2	*In vitro*	(123, 124)
**AP-1**	Extract of *Sigesbeckia orientalis*	Inhibits IL-1β, IL-6, IL-8, COX-2, MMP9, MAPKs	*In vivo*	(125)
**c-Jun**	Melittin	Inhibits COX-2, MMP1, MMP3, MMP8 and MMP13	*In vitro*	(106)

#### 4.3.2 Natural compounds

While there are no FDA-approved AP-1 inhibitors available, many studies have explored natural compounds that can potentially block components in the AP-1 pathway (**Table 3**). Antcin K is an extract taken from a medicinal mushroom, *Antrodia cinnamomea*, that inhibits vascular cell adhesion molecule 1 (VCAM-1) and monocyte adhesion to RASFs by inhibiting the phosphorylation of p38 and MEK1/2-ERK (122). Apigenin-4^´^-O-α-L-rhamnoside, a natural flavonoid, exhibits inhibitory mechanisms against MMP1, MMP3, TNF, and RNAKL in RA FLS by inhibiting the MAPK/JNK/p38 pathway (102). Anticin K and Apigenin-4^´^-O-α-L-rhamnoside inhibit the inflammatory mediators of AP-1 and indirectly suppress the activation of AP-1. Treatment with resveratrol directly suppresses bradykinin-mediated AP-1 and NF-κB activities and inhibits COX-2 production in RASFs (40). Melittin, the primary component of bee venom, exhibits inhibitory properties by suppressing MMP1 and MMP8 by blocking the phosphorylation of PI3-K/AKT and ERK/JNK and the translocation of c-fos (106). Thymoquinone is another natural compound found in *Nigella sative*, which shows anti-inflammatory properties in preclinical arthritis models, blocking multiple pathways that include AP-1 and NF-κB (127). The ethanolic extract of *Sigesbeckia orientalis* inhibits pannus formation and reduces cartilage damage and bone erosion in the collagen-induced arthritic model, while it leads to decreased expression of IL-1β, IL-6, IL-8, COX-2, MMP9, and NLRP3 by inhibiting MAPKs, AP-1, and NF-κB in *in vitro* studies carried out in synovial cells (125).

## 5 IRFs

### 5.1 Activation of IRFs

**Figure 4:**
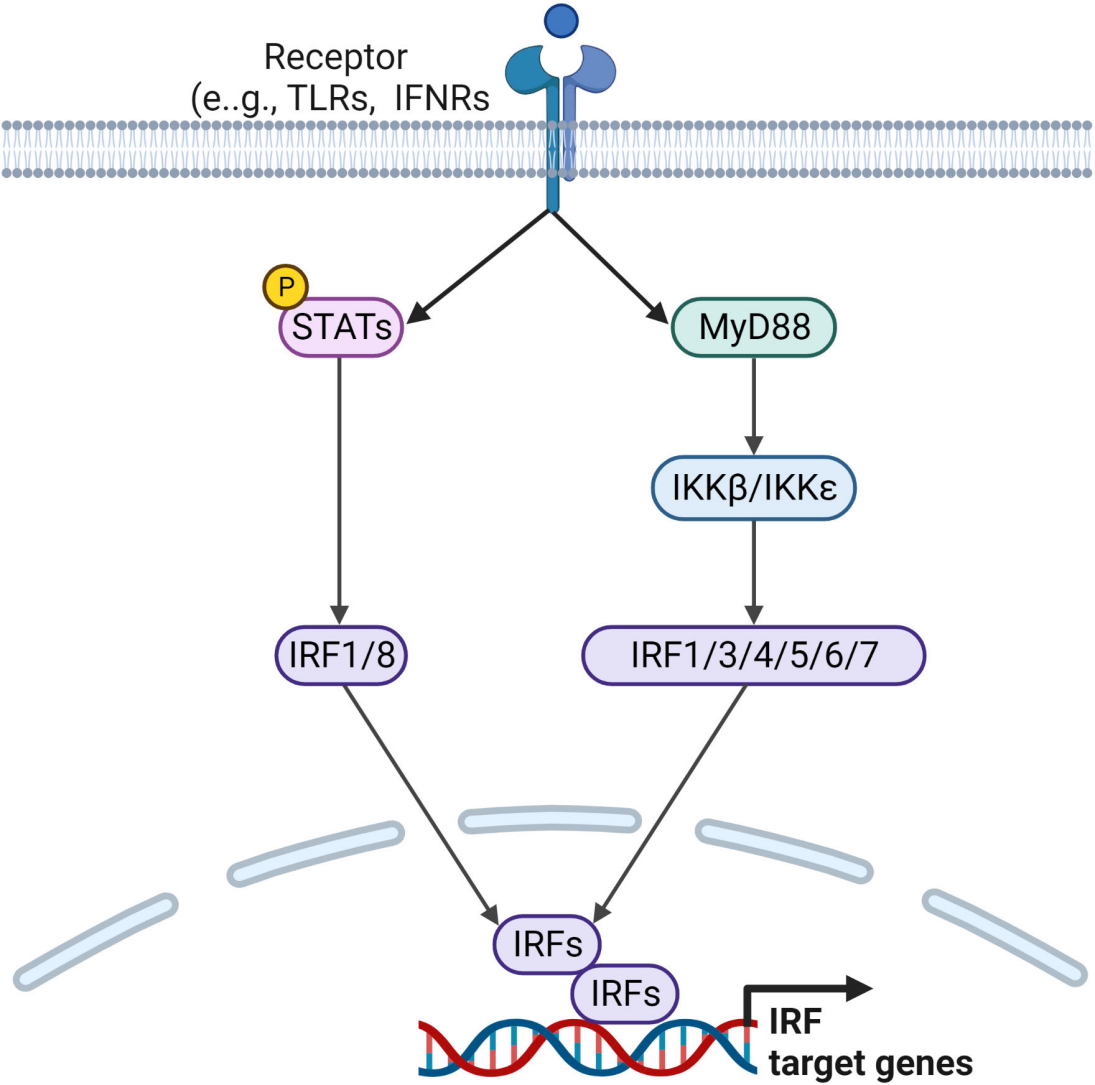
Signaling pathways leading to the regulation of IRF target genes. Ligands binding to TLRs and IFNRs initiate downstream signaling pathways via downstream activators and adaptor molecules, such as STATs and MyD88, which lead to the activation of IRF family transcription factors. Activated IRFs form homo or heterodimers before being translocated to the nucleus, where they regulate transcription of IRF target genes.

In humans, the interferon regulatory factor (IRF) family of transcription factors consists of nine members, IRF1 to IRF9. They share a homology region found in the N-terminal DNA binding domain, which binds to the interferon stimulated response element (ISRE). The diverse C-terminal domain is unique to each member and binds to a wide range the proteins outside the IRF family. They are activated via signals received by activation of TLR and BCR (128). Upon activation, IRFs can form homo- or hetero dimers, which are translocated to the nucleus. Hyperactivated IRFs mainly produce IFNs and thus contribute to inflammatory diseases. Each member of the IRF family is regulated by a range of inflammatory mediators present in RA synovium and their mode of activation is discussed below (**Figure 4**).

#### 5.1.1 IRF1

TLR-activated TAK1 can induce IRF1 transcription via the RelA/p50 complex (129). Furthermore, the JAK/STAT signaling cascade has also been shown to induce the expression of IRF1 (130). The following studies have also demonstrated a similar pathway of IRF1 activation. Pristane induces autophagy in macrophages and can induce IRF1 activation by activating STAT1 (66), and IKKβ regulates IRF1 transcription in conventional type 1 dendritic cells (129).

#### 5.1.2 IRF3

IRF3 is activated by various signals received from intracellular receptors such as RIG-1, MDA5, TLR3, TLR4, and cytosolic double-stranded DNA (dsDNA) sensors (131). Activated TLR3 signals via TRIF to activate TBK1 and IKKε via TRAF3. dsDNA in the cytosol can trigger type 1 IFN through STING, which can activate IRF3 by stimulating its phosphorylation by activating TBK1. It is suggested that the C-terminal tail of the STING oligomer can recruit both TBK1 and IRF3 by binding to the IRF3 motif and delivers IRF3 to TBK1 (132). Finally, TBK1, along with IKKϵ, phosphorylate IRF3 to form dimers and then translocate to the nucleus (133).

#### 5.1.3 IRF4

IRF4 is induced by activation of BCR and CD40 in B cells, TCR in T cells, and TLR in macrophages. However, it is not activated by type 1 or type 2 IFNs. The receptor activation leads to activation of c-Rel, which binds to the promotor region of IRF4 to induce transcription. The promotor region contains Foxp3, STAT4, STAT6, and IRF4 binding sites, suggesting that IRF4 is capable of autoregulating its expression (134).

#### 5.1.4 IRF5

IRF5 activation is initiated upon binding of ligands to TLR7/8/9, wherein MyD88, IRAK1/4, and TRAF6 are recruited. Then autophosphorylation of IRAK4 activates TAK1 to phosphorylate IKKβ. Meanwhile, TRAF6 ubiquitinates IRF5, which is subsequently phosphorylated by IKKβ, thus forming homodimers. IRF5 homodimers are translocated to the nucleus to activate target genes (128).

#### 5.1.5 IRF6, IRF7 and IRF8

IRF6 is regulated via TLR2 in epithelial cells and TLR3 in keratinocytes (135). In keratinocytes, RIPK4 phosphorylates and activates IRF6 (136). IRF7 is activated through TLR3 and TLR7 via two different pathways. TLR3 is activated by dsRNA and phosphorylates IRF7 via TRIF, while TLR7 signals via MyD88-TRAF6 signaling, which is induced by ssRNA (137). IRF8 is activated in macrophages, dendritic cells, T cells, and NK cells. Binding of IFNγ to its receptor activates IRF8 by activating STAT1. IRF8 is also activated following stimulation with IFNα/β and LPS (138).

### 5.2 IRF-regulated genes

IRFs play a major role in autoinflammation and autoimmunity (139). IRF1, IRF3, IRF5 and IRF7 are important in the induction of type 1 IFN, where IRF4, IRF5 and IRF8 regulate the development of myeloid cells and play a crucial role in inflammatory responses (140). A wide range of studies have suggested a role for each IRF in inflammatory diseases, including RA.

#### 5.2.1 IRF1

IRF1 regulates several IFN-regulated genes (e.g., CXCL9, CXCL10, and CXCL1)1 in rheumatoid synovium and activates B cell activating factor (BAFF), which is highly expressed in RA (141). IRF1 regulates TNF-induced IFNβ expression and subsequently activates STAT1 to activate IFN-regulated genes (141). IRF1 can induce TLR3 expression in pristane-induced arthritis (66). A recent study demonstrated that the invasiveness of synovial fibroblasts is regulated by the expression of follistalin-like protein 1 induced by IRF1 (142).

#### 5.2.2 IRF2 and IRF3

IRF2 negatively regulates IFN type 1 signaling and counterbalances the activity of IRF1. IRF2 activates IL-12p40 and VCAM-1, leading to the development of NK cells and Th1 cells, respectively (143). IRF2 stimulates inflammatory ROS levels, TNF, IL-1β, and IL-6 expression, and suppresses superoxide dismutase. The knockdown of IRF2 gene is shown to inhibit the JAK/STAT signaling pathway (144). IRF3 regulates the expression of IL-6, IL-8, MMP3, and MMP9 in RA FLS by activating c-Jun/AP-1 (145).

#### 5.2.3 IRF4

IRF4 plays a diverse role in inflammation and arthritis. It is mainly involved in T cell differentiation. IRF4 responds to IL-4 and regulates Th1 and Th2 differentiation through interaction with T-bet and GATA3, respectively. IRF4 binds directly to RORγt and mediates the differentiation of Th17 cells (146) and regulates Glut1, IL-17 and IL-21 levels (147, 148). Furthermore, it interacts with BCL-6 and Foxp3 to produce T follicular helper cells and Tregs, respectively (146). In macrophages, IRF4 distorts macrophages into the M2 phenotype through JMJD3 competing for MyD88 with IRF5 while suppressing M1 polarization of M1 and inducing IL-4 and IL-10 secretion. Previous studies have shown that a pro-inflammatory cytokine GM-CSF can regulate CCL17 formation in monocytes/macrophages through JMJD3 and IRF4 (78). In addition to these functions, IRF4 binds to STAT3, STAT6, and NFATs to carry out transcription. IRF4 functions as a transcriptional repressor by forming a homodimer or heterodimer with IRF8, suppressing the expression of IFN-inducible genes and inhibiting IRF1 activity in macrophages and T cells (134).

#### 5.2.4 IRF5

Studies show that IRF5 acts in T cells, monocytes, and macrophages. Increased expression of IRF5 induces M1 polarization while suppressing M2 polarization (128). IRF5 increases the expression of IL-12 in circulating monocytes in samples from OA patients without treatment and promotes Th1-related genes in resting T cells (149). Furthermore, IRF5 induces a wide range of pro-inflammatory cytokines such as IL-17, monocyte chemotactic proteins (MCP-1), TNF-α, RANTES, IL-6, IL-12p40, and IL-23p40 (150, 151). IRF5 up-regulates MMP3 production mediated via NF-κB (151).

#### 5.2.5 IRF6 and IRF7

TLR3-induced activation of IRF6 leads to enhanced expression of IL-23p19, while negatively regulating IFNβ expression by competing with IRF3 in the IFNβ promotor region or by forming a heterodimer complex with IRF3 (135). In addition, IRF6 induces the expression of IL-8, CCL5, and CXCL11 (136, 152) and IRF7 mediates RANKL production in RA FLS (137).

#### 5.2.6 IRF8

IRF8 is crucial for the development and maturation of myeloid cells. At the transcription level, IRF8 is co-recruited to form ternary complexes with other TFs. It forms a heterodimer with IRF1, STATs, AP-1, and PU.1 (138, 143) and induce the production of IL-6, IL-12p40 and TNF. On the other hand, IRF8 negatively regulates osteoclastogenesis by inducing IFNγ (153). Recently, it was found that IRF8 can promote the expression of MMP13 in OA (154).

### 5.3 Current treatments targeting IRFs

Since the IRF family of TFs is involved in the regulation of a wide range of inflammatory mediators, they can be potential treatment targets for RA. Currently a type 1 IFN inhibitor, anifrolumab, is subjected to phase 2 clinical trials (155). A study in RA patients by Juge et al. shows a IRF5 response to rituximab within 24 weeks (156). Certain JAKi, such as tofacitinib and baricitinib, are documented to suppress the activity of certain IRFs by inhibiting STAT1 activity (141).

## 6 Other TFs

In addition to the TFs discussed above, other TFs such as, hypoxia-inducible factor (HIF) and nuclear factor-erythroid 2-related factor-2 (Nrf2), are also implicated in the pathogenesis of RA. In RA synovium, HIF is activated during hypoxia, which aggravates angiogenesis, synovial hyperplasia, and pannus formation (157–159). TNF, IL-1β, and IL-33 can induce the expression of HIF in RASFs and resident macrophages (157). Production of HIF primarily induces the expression of VEGF, which promotes the synthesis of proteolytic enzymes in endothelial cells (160). Furthermore, it promotes the generation of M1-type macrophages and Th17 cells (161, 162). Knockdown of HIF-α in collagen-induced arthritis (CIA) mouse model has been shown to inhibit multiple inflammatory pathways and thereby, ameliorating arthritis (163). In recent years, several studies are focusing on HIF inhibitors as potential therapeutics for treating arthritis. Pharmacological HIF inhibitor, PT2977 has been shown to ameliorate arthritis in the CIA mouse model (164). Moreover, natural compounds, that include andrographolide, geniposide, dihydroarteannuin, and tylophorine-based compounds, can inhibit HIF and be effective in attenuating RA progression (163, 165–167).

Nrf2 is a redox regulator, which plays a protective role by exerting anti-inflammatory and antioxidant effects (168). Significantly, the protective role of Nrf2 has been linked to relieving severe symptoms in RA via detoxification, regulation of redox balance, and metabolism (34, 169). TNF and increased ROS levels can induce the expression of Nrf2 in RA synovium, which in return suppresses the proliferation and MMPs production in RAFLS via inhibition of inflammatory mediators activated in RA (169). Due to this protective role of Nrf2, studies are focusing on synthetic drugs, including, dihydroartemisinin, and dimethyl fumarate, as well as natural compounds, including sinomenine, licochalcone, 7-deacetyl-gedunin, calycosin and resveratrol, that increase the expression of Nrf2 to treat RA (170–174; 90). While both HIF and Nrf2 have been identified as potential treatment targets for RA, further studies, utilizing the above-mentioned synthetic and natural compounds, are required to explore their therapeutic potential.

## 7 Network of TFs

In a complex disease, such as in RA, all the aforementioned TFs can form a network to cross-regulate each other or function cooperatively to activate or antagonize downstream target genes (175, 176). Examples of such interactions are STAT1-STAT2 with IRF9, STAT1 with NF-κB, STAT3 with Jun, STAT3 with IRF4, RORγt, and BAFT in T cells, and STAT1 with IRF1, IRF8 and PU.1 in macrophages (146, 177). Further, c-Fos/AP-1 and NFATc1 together control the osteoclast differentiation (110). IRF4 can bind to STAT3, STAT6, and NFATs to facilitate transcription of their downstream genes but it can also function as a transcriptional repressor by forming a heterodimer with IRF8, suppressing the expression of IFN-inducible genes and inhibiting IRF1 activity in macrophages and T cells (134). AP-1, NF-κB, and IRFs together are known to activate MMPs, (178). NF-κB, IRF4/8, PU.1, AP-1, and STAT1 induce the expression of IL-1β (179). STAT3 can activate HIF in RA synovium (180). While these studies highlight the complex network of TFs and their regulation of downstream inflammatory mediators, a careful approach is warranted when targeting them for therapeutic benefits. Since these TFs function both individually and cooperatively, targeting one or more TFs can effectively ameliorate RA. However, the key TFs involved in RA pathogenesis are also associated with biological processes involving homeostasis, and therefore inhibiting these TFs may lead to undesirable side effects. This challenge is currently being addressed by tissue-specific/joint-specific drug delivery via nanocarriers, which increase the specificity and efficacy, while minimizing potential adverse effects (17, 30, 31, 35, 121, 181–183).

## 8 Conclusion and prospect

RA is a chronic inflammatory autoimmune disease, causing pain and disability. Several drugs that are currently used for RA treatment are effective only delaying the progression of the disease or alleviating inflammatory symptoms. Many of these drugs have drawbacks, including disease recurrence and adverse effects due to long-term use. Therefore, there is a need to develop novel therapeutic strategies to address these shortcomings.

TFs play important roles in immune and nonimmune cells through regulation of gene expression. Studies emphasize the importance of the forementioned TFs in RA disease initiation and progression of RA disease. With the approval of JAK inhibitors in the treatment of RA, the pursuit of TFs or their signaling pathways as potential treatment targets has gained momentum. Currently, several inhibitors of TFs are being investigated, and they block TF function by inhibiting protein-protein interaction, translocation of TFs from the cytosol to the nucleus, or protein-DNA binding.

In summary, this review highlights key TFs and their signaling pathways that may become targets for future RA therapies; it also provides an update on several synthetic drugs and natural compounds that are in consideration for targeting such TFs or the signaling pathways that activate TFs.

## Author contributions

All authors contributed to the writing and revision of the manuscript. TB and AA developed the concept, structure, and prepared tables and figures. TB drafted the original manuscript. AA, JH, and KL reviewed and edited.
